# Molecular Phylogenetic Relationships of Flightless Beetles Belonging to the Genus *Mesechthistatus* Breuning, (Coleoptera: Cerambycidae) Inferred from Mitochondrial COI Gene Sequences.

**DOI:** 10.1673/031.008.7001

**Published:** 2008-11-14

**Authors:** Hiroshi Nakamine, Makio Takeda

**Affiliations:** ^1^ Sanda Municipal Arimafuji Nature Study Center, Fukushima 1091-2, Sanda, Hyogo 669-1313, Japan; ^2^ Kobe University, Graduate School of Science and Technology Rokko-dai 1-1, Nada, Kobe, Hyogo 657-8501, Japan

**Keywords:** longicorn beetle, phylogeography

## Abstract

The longicorn beetles belonging to the genus *Mesechthistatus*Breuning., 1950 (Coleoptera: Cerambycidae) cannot fly since their hindwings are atrophied. This slows down gene flow between local populations. Currently, it is considered that the genus contains four endemic species from the eastern Honshu Is., Japan, *M. binodosus, M. furciferus, M. taniguchii* and *M. fujisanus*, that are distributed parapatrically. Sequence analyses of the cytochrome oxidase subunit I gene suggests that lineages of mitochondrial haplotypes split approximately in the same era. However, this result is not consistent with the monophyly of morphological species. The estimated evolutionary rate of the COI gene in other insects suggests that mitochondrial haplotypes of *Mesechthistatus* differentiated at the end of the Pliocene epoch during the Tertiary era.

## Introduction

**Figure 1.**
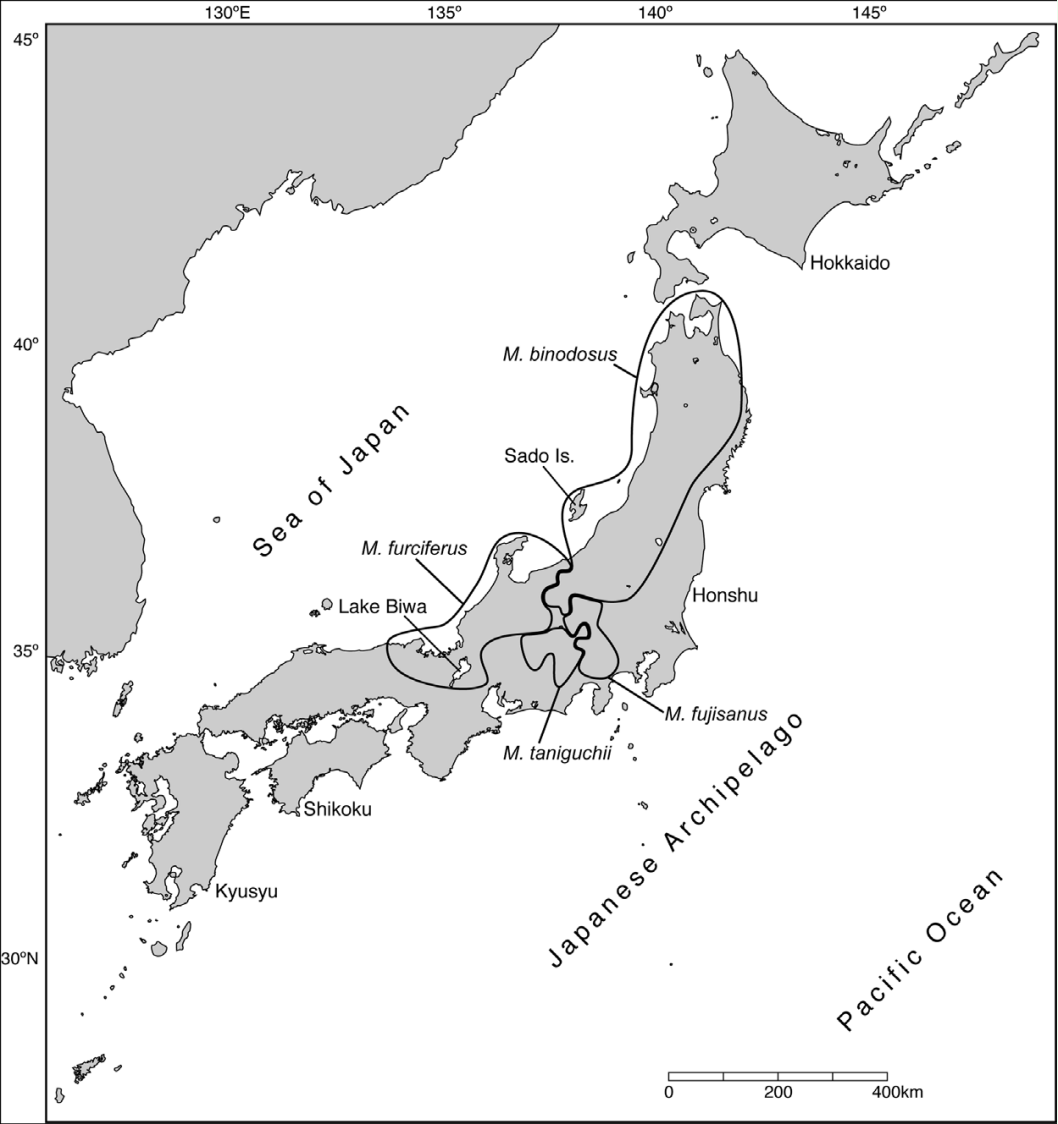
Japan showing the distribution areas of four *Mesechthistatus* species.

Situated at the eastern tip of the Eurasian plate, the Japanese Archipelago forms an island arc with a total length of 3000 km (Figure 1). The landmass was separated from the Eurasian continent between 20 and 16 million years ago (mya) during the Miocene epoch of the tertiary period of the Cenozoic era and became the basic elements of the present day archipelago (Maruyama et al. 1997; Otofuji et al. 1985, 1996). Subsequently this land sank, and the land started to rise in the west, resulting in the formation of the archipelago around 5 million years ago in the Pliocene epoch (Maruyama et al. 1997). More recent studies (Kitamura et al. 2001; Kitamura and Kimoto 2006) reported that the Japanese Archipelago was connected to the East Asian continent between 3.5 and 1.7 mya except for a few short periods of isolation lasting 10,000 to 20,000 years. After 1.7 mya, in the Quaternary era, it is considered that unstable land bridges were constructed several times between the Japanese Archipelago and the Continent at glacial maxima (Kawamura 2007; Shinohara et al. 2004; Suzuki et al. 1997, 2003; Tsuchiya et al. 2000). These caused geographic links and separation from the continental biome, and subsequent colonization of the archipelago completed the speciation process between the early and late colonizers in the archipelago. In addition, the rising land level in the period after the Quaternary era in the central region of Honshu resulted in complicated topography. For animals of low mobility, high mountains and other geographical features became barriers to migration, and it is thought that separation and speciation occurred at regional population levels.

These events in the geological history formed a diversity of endemic species in the Japanese Archipelago. For example, an extensive taxonomic survey described 755 species of Cerambycidae, of which 463 are endemic to Japan (Ohbayashi and Niisato 2007). Investigations to the subfamily level gave the following figures (the number of endemic species/the number of total species in the Japanese Archipelago): Parandrinae; 1/2, Prioninae; 4/13, Spondylidinae; 4/15, Lepturinae; 118/157, Necydalinae; 7/10, Cerambycinae; 97/198, Lamiinae; 232/ 360 with notably high numbers of endemic species in Lepturinae and Lamiinae.

**Figure 2.**
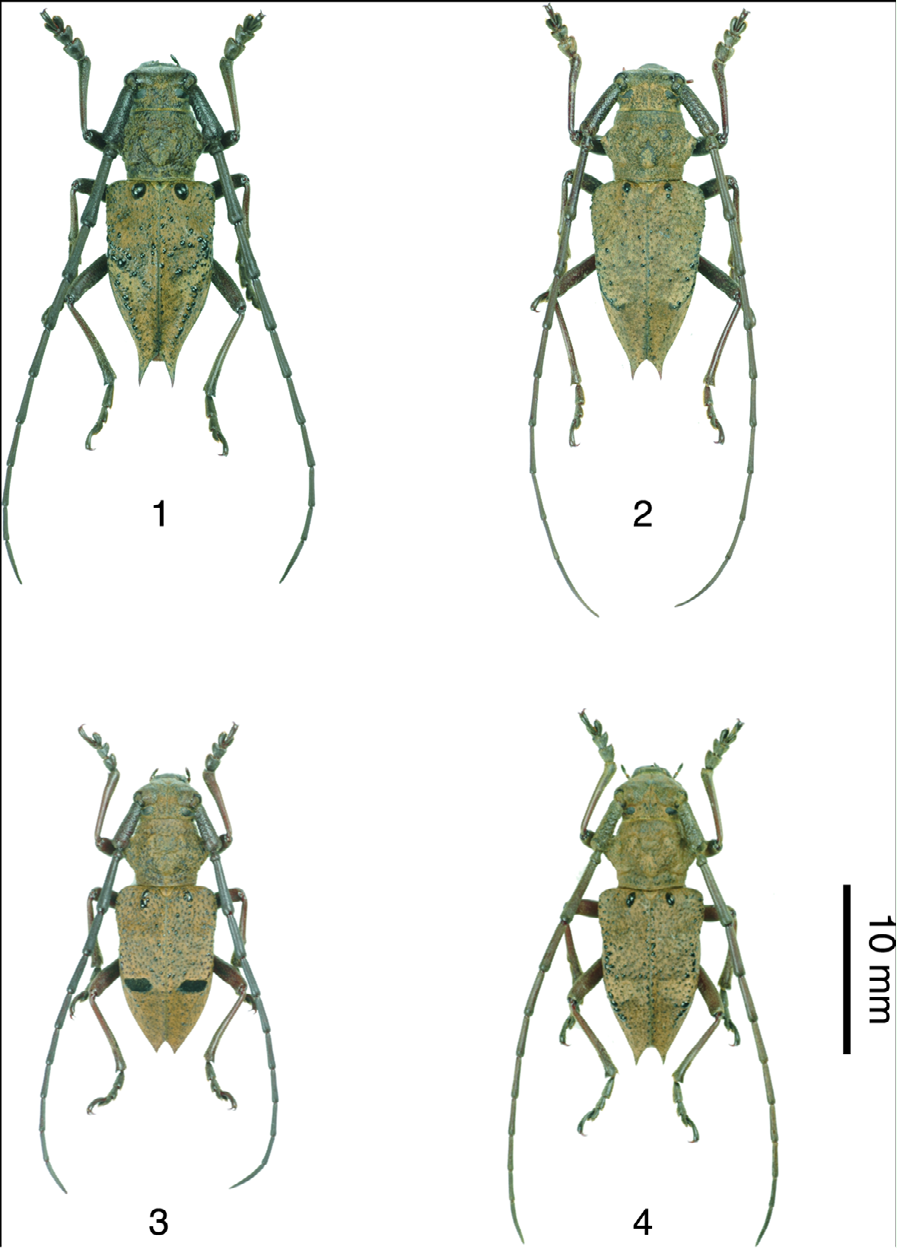
Photographs of *Mesechthistatus* spp. 1, *M. binodosus*; 2, *M. furciferus*; 3, *M. taniguchii*; 4, *M. fujisanus*

The genus *Mesechthistatus*Breuning, 1950 discussed in this paper belongs to the tribe Phrissomini Thomson, 1860 and the subfamily Lamiinae Latreille, 1825, and contains four species based on morphology and distribution patterns: *M. binodosus* (Waterhouse), *M. farciferus* (Bates), *M. taniguchii* (Seki, 1944), and *M. fujisanus* Hayashi, 1957 (Figure 2). *Mesechthistatus yamahoi* (Mitono, 1943), previously described in Taiwan (Mitono 1943), also belongs to this genus. However, with no subsequent records since the time of original entry, the existence of this species is currently in doubt (Hasegawa 2007). *Mesechthistatus* is a genus endemic to Japan, and is found only from Honshu and Sado Island. The above-mentioned four species have parapatric distribution (Figure 1). The atrophied hindwings common to all species render them incapable of flight, and thus their mobility must be limited. As a result, morphological disparities have been found, with two subspecies for each of the species described as *M. binodosus* and *M. furciferus* (Hayashi 1951, 1955).

In this paper, to investigate interspecific differences of *Mesechthistatus* and to estimate the time of colonization in Japanese Archipelago, molecular phylogenetic analysis was carried out using partial sequences from the mitochondrial cytochrome oxidase subunit I gene from four different species of *Mesechthistatus*.

## Materials and Methods

### Taxon sampling

**Figure 3.**
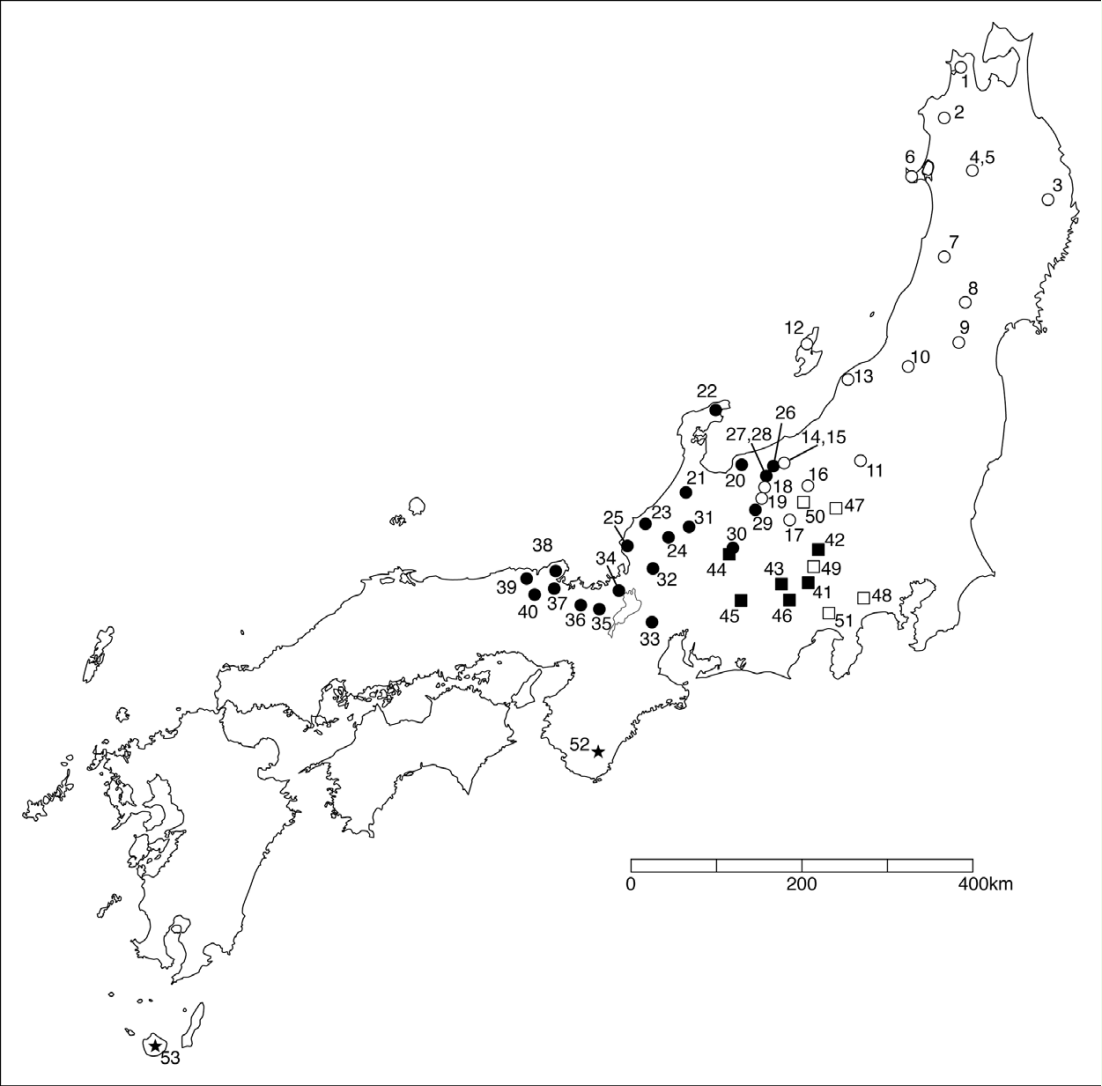
The localities where specimens of four *Mesechthistatus* species were collected. Open circle, locality of *M. binodosus* specimens; closed circle, locality of *M. furciferus* specimens; open square, locality of *M. furciferus* specimens; closed square, locality of *M. taniguchii* specimens; closed star, localities of *Porechthistatus* and *Hayashiechthistatus* used as outgroup specimens for phylogenetic analysis. Locality numbers correspond to the number in Table 1 and the phylogenetic trees in Figure 4.

The specimens of *Mesechthistatus* spp. analyzed in this study are listed in Table 1, and the localities where they were collected are shown in Figure 3. The beetles were immediately fixed in 95–99.5% ethanol and preserved in the same solution until use. A single individual from each locality was used for DNA extraction.

### Extraction, PCR amplification and sequence analysis of DNA

Total DNA was extracted from a mixture of cephalic and thoracic muscles by using a GenElute™ Mammalian Genomic DNA Miniprep Kit (Sigma-Aldrich Inc., www.sigmaaldrich.com). Each DNA sample was dissolved in 200 µ″1 elution buffer. A fragment of DNA encoding mitochondrial cytochrome oxidase subunit I was amplified from the total DNA solution using PCR with a primer pair as follows: KobCI1.2 (5′-TAA GAA GAA TTG TAG AAA ATG G-3′) and YhzCI2.2 (5′-TGT AGC GAT TTC TAA AAA AAGG-3′). PCR was carried out in a 25 µ″1 reaction mixture containing 1 X PCR buffer for KOD -Plus- (Toyobo Biologies Inc., www.toyobobiologics.com), 0.2 mM of each dNTP, 2 mM of MgSO4, 0.5 unit of KOD -Plus- DNA Polymerase (Toyobo), 0.3 µ″M of each primer and 1 µ″1 of template DNA solution. The amplification protocol was 25 cycles of denaturation at 94°C for 15 sec, annealing at 50°C for 30 sec, and extension at 68°C for 40 sec in a GeneAmp® PCR System 9700 (Applied Biosystems, www.appliedbiosystems.com). The PCR product was purified using by a GenElute™ PCR Clean-Up Kit (Sigma-Aldrich Inc.). Direct sequencing of the COI DNA fragment was performed by using a BigDye® Terminator Cycle Sequencing Kit (Applied Biosystems) with primers KobCI1.2 and Yhz2.2. A partial sequence of the COI gene was determined by a ABI PRISM® 310 Genetic Analyzer or ABI PRISM® 3100 Genetic Analyzer (Applied Biosystems).

### Phylogenetic analysis

The sequence alignments were performed by using CLUSTAL W version 1.83 (Thompson et al. 1994), and no indels (insertions/deletions) were required for the alignments of COI sequences. A transition/transversion ratio R was obtained with PAUP*version 4.0b10 (Swofford 2002).

Phylogenetic tree topology was constructed according to the maximum likelihood (ML) method in PAUP^*^ version 4.0b10. The ML model was selected by hierarchical likelihood ratio tests (hLRTs) and akaike information criterion (AIC) using MrModeltest version 2.2 (Nylander 2004) with PAUP^*^ version 4.0b10, and heuristic searches were performed using the GTR+I+G model of the substitutions with TBR branch-swapping algorithm. The starting tree was obtained via stepwise addition, and the starting branch lengths obtained using Rogers-Swofford approximation method. The bootstrap test was executed on 200 replicates using GARLI version 0.951 (Zwickl 2006).

### Estimation of divergence time

To estimate divergence time, PATHd8 was used (Britton et al. 2007). The PATHd8 is a program for phylogenetic dating without a molecular clock. First, a ML tree topology was prepared using PAUP^*^ version 4.0b10 with the GTR+I+G model. Next, branch lengths were obtained using an estbranches program that included the multidistribute program package (Thorn et al. 1998) with PAML (Yang 1997). Thus, we prepared input file (tree with branch lengths in Newick format) for PATHd8.

One constraint was used on one node. *Mesechthistatus binodosus* is distributed in Honshu and Sado Is. (Figure 1). Oshima (1990) estimated that a channel separated Sado Island and Honshu between 0.8 and 0.2 Mya. The obtained likelihood branch length between “BIN 12” collected from Sado Island and “BIN 8” collected from Honshu, are the shortest. Therefore, 0.8 mya was used as the upper fix age and 0.2 mya was used as the lower fix age at the node of “BIN 8” and “BIN 12”.

## Results and Discussion

### Genetic data

A partial sequence of 1144 base pairs of mitochondrial COI gene was obtained from *Mesechthistatus* spp. In this study, throughout the COI sequences used, neither deletions nor insertions were found in multiple alignment. Altogether 236 sites were variable including 33 at the first position, 4 at the second position and 189 at the third position. The transition/transversion ratio R was 5.103. The base frequencies were nearly constant among four *Mesechthistatus* spp. as follows: A, 0.309971; C, 0.163393; G, 0.149304 and T, 0.377331. These data suggest an A-T bias.

**Table 1.**
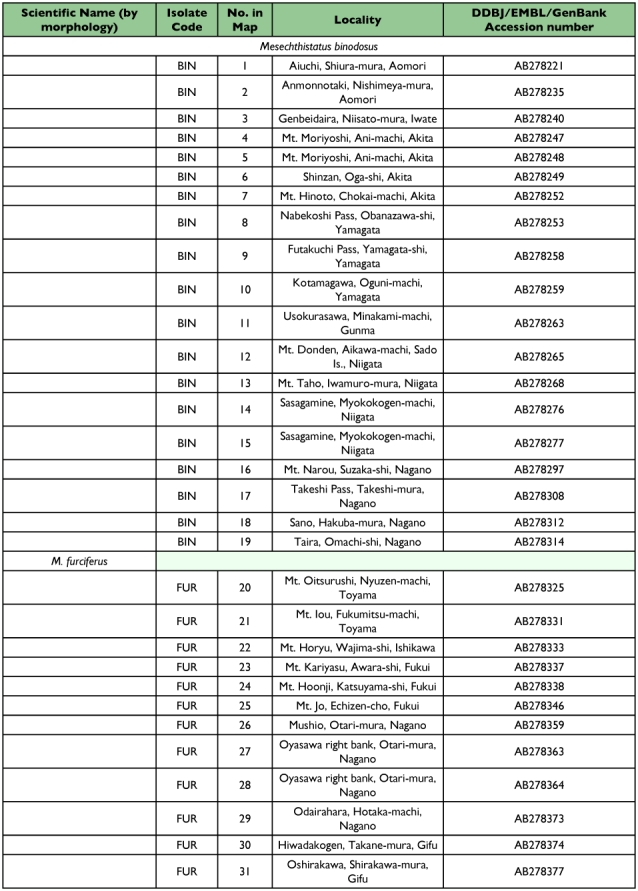
The specimens analyzed in this study.

**con't:**
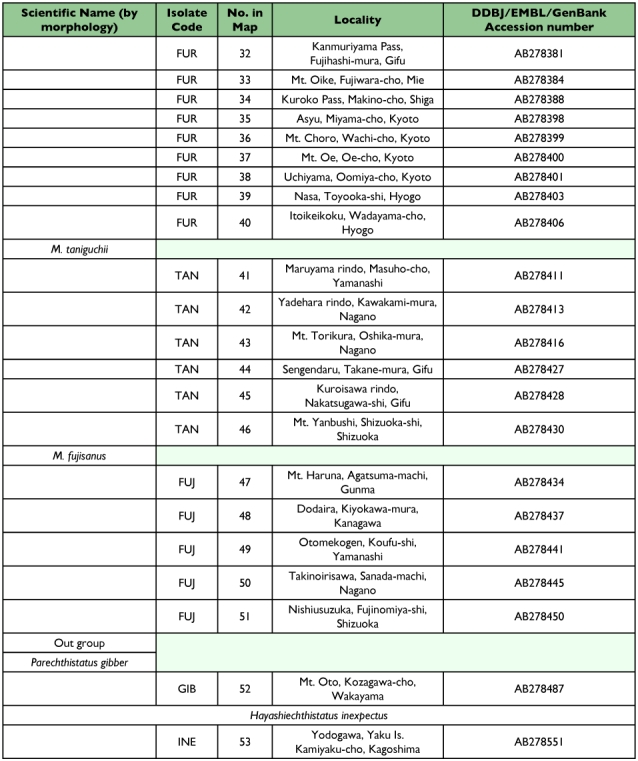

### Molecular phylogenetic analysis

**Figure 4.**
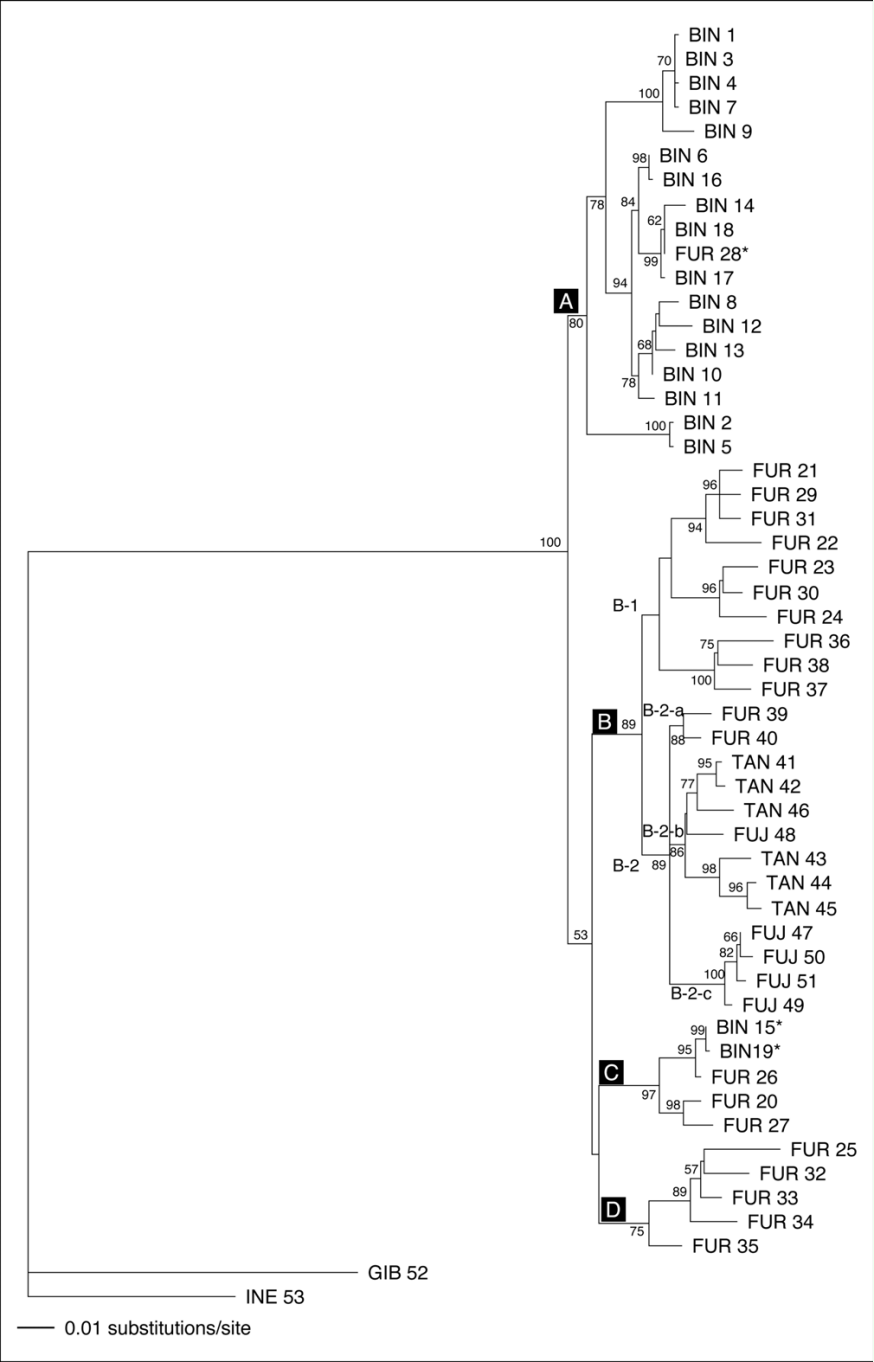
Maximum likelihood tree based on the mitochondrial COI gene sequences of the *Mesechthistatus* spp. The bootstrap value was indicated at each node (when > 50%). The numbered specimen identity corresponds to the number for locality in Table 1, Figure 3 and Figure 5. Asterisk indicates introgressive specimens.

**Figure 5.**
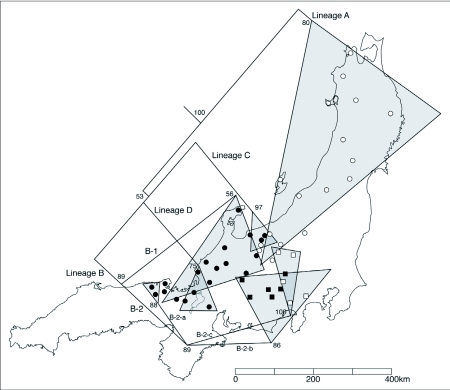
A simplified tree showing relationship between major lineage and geographic distribution in the study. The letter beside each lineage line indicates correspondence to the letter in Figure 4. The bootstrap value was indicated at each node (when > 50%). Open circle, locality of *M. binodosus* specimens; closed circle, locality of *M. furciferus* specimens; open square, locality of *M. fujisanus* specimens; closed square, locality of *M. taniguchii* specimens.

Figure 4 shows the maximum likelihood phylogenetic tree. Basically four lineages of ancestry were recognized, as marked A, B, C and D. A schematic diagram for relatedness of the 4 lineages is shown in Figure 5.

Lineage A: Lineage A contained a single species, *M. binodosus*, showing the monophyletic origin of the *M. binodosus* COI haplotype (Figure 4). However, morphological characteristics and the fact that the location of collection was within that of the *M. furciferus* distribution area imply that *M. furciferus* (FUR 28) should be included in linage A. As a specimen of *M. furciferus* was collected in the vicinity of the *M. binodosus* distributional boundary, it is likely that introgressive hybridization (e. g. Nagata et al. 2007; Shimizu and Ueshima 2000; Sota et al. 2001) took place from *M. binodosus* to *M. furciferus* sometime in the past, thus giving “FUR 28” the *M. binodosus* haplotype. Given that the “FUR 27” that was collected at the same location as “FUR 28” has the same COI haplotype as *M. furciferus*, it is likely that individuals that underwent introgressive hybridization now exist together as a single group in one location.

Lineage B: This lineage was not monophyletic but complex, including 3 species: *M. furciferus, M. tanguchii*, and *M. fujisanus*.

B-1 contains a single species, *M. furciferus* and shows a unique distribution pattern (Figure 5). Lineage D is distributed in the gap zone between the B-1 populations since it has another ancestral mitochondrial haplotype. Lineage D is considered to have diverged at an earlier time than the lineage B, and it is possible that the mitochondrial haplotype of lineage D expanded its distribution into the area of lineage B distribution. Lineage B diverged into several sublineages thereafter.

*M. furciferus, M. taniguchii*, and *M. fujisanus* form the B-2 sublineage. The B-2-a sublineage is made of populations found in the vicinity of the western limit of the *M. furciferus* distribution. The B-2-b sublineage is essentially made of *M. taniguchii* showing monophyletic origin, though an individual “FUJ 48” was recognized morphologically as *M. fujisanus*. Until now, neither *M. taniguchii* nor *M. fujisanus* has been found to possess a mitochondrial haplotype closely related to the haplotype found in this sample. This suggests introgressive hybridization between *M. taniguchii* and *M. fujisanus* at some time in the past. Sublineage B-2-c includes *M. fujisanus*, and the genetic disparity among populations was small. Possible explanations for this include the emergence of a bottleneck and rapid distribution expansion at some time in the past.

Within the B-2 sublineage, there is a contiguity in the distribution areas of B-2-b (*M. taniguchii*), and B-2-c (*M. fujisanus*). However, the distribution area of the B-2-a population occurs at the western border of *M. furciferus*, and it is isolated from the other lineage by a considerable distance. *Mesechthistatus* has not been found in the southwest region of Honshu, Shikoku and Kyushu, although the genus *Parechthistatus*, a member of the tribe that *Mesechthistatus* belongs to, inhabit that region. It is possible that the groups with the mitochondrial haplotype found in the B-2-a became fragmented due to the distribution expansion of *Parechthistatus*.

Lineage C: *M. furciferus* and *M. binodosus* form the lineage C from an extremely small area in the northern part of the Chubu region (Figure 5). It is considered that this lineage originally possessed the *M. furciferus* mitochondrial haplotype, though “BIN 15” and “BIN 19” are included in this lineage. As this population is found in the vicinity of the distribution boundary, it is likely that *M. furciferus* mitochondrial DNA was introgressed into *M. binodosus*.

Lineage D: *M. furciferus* forms a single lineage D, distributed around Lake Biwa (Figure 5).

### The divergence date of *Mesechthistatus* spp

For molecular phylogenetic analysis, estimation of the evolutionary rate of sequence divergence is of utmost importance as a molecular clock. The estimation of the final divergence time based on this rate is a major goal. However, there is little knowledge of geological events and a lack of fossil records for calibration of the chronological relation between phyletic lineages and genetic distances in the *Mesechthistatus* COI gene. Therefore, it is difficult to exactly estimate the rate of evolution of the COI gene in this study. However, the calibration of the evolutionary rate has been estimated based on COI gene in numerous other insects, giving an estimate ranging from 1.5% per 1 million years in *Tetraopes*: Cerambycidae (Farrell 2001) and *Crematogaster*. Formicidae (Quek et al. 2004), 1.6% in *Plateumaris*: Chrysomelidae (Sota and Hayashi 2007) to 2.3% in *Heliconius*: Nymphalidae (Brower 1994). The date of radiation in *Mesechthistatus* was estimated by using these values.

Figure 4 depicts four major lineages, A to D. The average value for genetic divergence between the four lineages was 4.53 ± 0.45 (mean ± SD) %. Application of the evolutionary rate of the COI gene (1.5% to 2.3%) dates the divergence timing of the major four lineages between 3.02 and 1.96 mya.

The divergence times were also estimated using the PATHd8 program that is not based on a molecular clock. The divergence time at the root node was between 5.44 and 1.36 mya, in the constraints that Sado Island was formed between 0.8 mya, as the upper fix age, and 0.2 mya, as the lower fix age, which corresponds to the distance for node of “BIN 8” and “BIN 12”. This result does not contradict the estimated times indicated above.

It is probably appropriate to assume that the ancestor of *Mesechthistatus* colonized from the East Asian continent, because *Parechthistatus chinensis*Breuning, 1942 that was related to *Mesechthistatus*, has been recorded in the Shaanxi Province of the People's Republic of China (Breuning 1942). Therefore, it is considered that colonization by *Mesechthistatus* of the Japanese Archipelago occurred in the late Pliocene epoch. The Japanese Archipelago was connected to the East Asian continent between 3.5 and 1.7 mya (Kitamura et al. 2001; Kitamura and Kimoto 2006).

Sota and Hayashi (2007) reported colonization history of *Plateumaris* Thomson, 1859 leaf beetles in Japan. They estimated the timing of colonization by using the Bayesian approach based on fossil records calibrations. They concluded that *Plateumaris constricticollis* (Jacoby), a Japanese endemic species, was formed before the late Pliocene.

Therefore, these results and other reports suggest that *Mesechthistatus* underwent radiation at the end of the Pliocene epoch. In order to further investigate the evolution of speciation in *Mesechthistatus*, it will be necessary to conduct a comprehensive molecular phylogenetic analysis that includes nuclear DNA.

### Editor's note

Paper copies of this article will be deposited in the following libraries. Senckenberg Library, Frankfurt Germany; National Museum of Natural History, Paris, France; Field Museum of Natural History, Chicago, Illinois USA; the University of Wisconsin, Madison, USA; the University of Arizona, Tucson, Arizona USA; Smithsonian Institution Libraries, Washington D.C. USA; The Linnean Society, London, England.

## Abbreviations

**COI**: cytochrome oxidase subunit 1,
**ML**: maximum likelihood,
**mya**: million years ago
